# A Case of Cataract Complicated with Tremulous Iris, Successfully Treated

**Published:** 1844-01

**Authors:** Wm. M. Boling

**Affiliations:** Montgomery, Alabama


Art. II.—*A Case of Cataract Complicated with Tremulous Iris, successfully treated.* By Wm. M. Boling, M.D., of Montgomery, Alabama.

The following case of "Tremulous Iris" combined with cataract, I am induced to offer for publication, from meeting with the following passage in Lawrence's Treatise on the Eye. Speaking of the preternaturally fluid state of the vitreous humour, on which the tremulous iris depends, he says:—"Usually this state of the vitreous humour indicates a diseased condition of the internal parts of the eye—and we find that the retina has lost its sensibility in such cases. Such a state of the vitreous humour cannot be remedied, *and if a catarat be present, its removal will not improve vision.*"

Isaac, a negro slave, between fifty-five and sixty years old, of a large and powerful frame, and good general health, twenty-five years ago become entirely blind, soon after which, his left eye was operated on for cataract. A high degree of inflammation followed the operation, and any improvement in his vision was for a long time despaired of. The inflammation, however, subsided, and gradually his sight improved, so that at the end of a year a very useful degree of vision was obtained. He recently changed masters, and his new one, anxious to have his sight improved, if possible, brought him to me for that purpose.

May 29th, 1843.—His eyes presented the following appearance. The pupil of the left eye was clear, and a good deal dilated. The slightest motion of the head caused the free margin of the iris to float about in a very peculiar manner. A slight movement of the head to the right would cause the left or outer portion of its free margin to become narrowed, or folded on itself so as to appear narrowed—reverse the motion, and the other side would assume the same appearance. Throw the head back and its margin would float forward—or throw the head forward and it would float back. The pupil was almost entirely uninfluenced by light, yet the vision was tolerably good, but had failed some within the last year. In the right eye was a cataract of a white color. The pupil was very much contracted. The iris had the same tremulous motion, but its movements were only backward and forward. This was owing to adhesions between the pupillary margin of the iris and the capsule of the lens. The application of belladonna produced scarcely a perceptible degree of dilatation, but caused the margin of the pupil to have a ragged, uneven appearance. No better effect was produced by the application of the bruised leaves of the stramonium, for several hours to the eye. He had a slight perception of light with this eye, when placed in a darkened room, with one open window, the eye directed towards it.

June 2d.—I operated by breaking up the cataract, which proved to be very soft. After the introduction of the needle, I discovered that the capsule was opaque, and became fully satisfied respecting the adhesion between it and the iris. I separated, completely as I thought at the time, the adhesions, and depressed the capsule, together with some of the larger portions of the lens below the axis of vision. Some small portions of the lens fell into the anterior chamber. Immediately on the separation of the capsule, the pupil became largely dilated. On the withdrawal of the needle, which was much more delicate than the cataract needles in common use, a very perceptible quantity of the vitreous humour followed it. I closed the eye immediately, and did not allow him to open it for several days. The usual means to prevent inflammation in such cases, were resorted to, and so little inconvenience did he suffer, that happening to call about a week after the operation, I found that he had gone out of his own accord to attend to his usual occupations. I made him return to his room—but from the 20th of June, he required no further attention.

Calling this morning (October 20th, 1843), to examine his case, I find the pupil of the right eye more contracted than that of the left. The *lateral* motions of the iris are now present in the right eye as in the left. A very narrow crescent-shaped portion of the opaque capsule is apparent, bordering the upper fourth of the circumference of the pupil. The rest of the pupil is quite clear. His vision out of the right eye (which is still less perfect than that of the left eye) is such, that, without glasses, he can readily count the number of fingers, or distinguish a pencil, watch-key, penknife, &c., when held up before him.

This case proves that a preternaturally fluid state of the vitreous humour, with tremulous iris, does not in *all cases* imply that the eye is incapable of a very useful degree of vision, or that the removal of a cataract under such circumstances would be entirely useless. The appearance of the iris in such cases is singular and striking, and would seem to depend on something more than a want of support from a softened state of the vitreous humour. To my mind it conveys the idea of paralysis, and this idea is supported by the imperfect manner in which the iris responds to light.

October 20th, 1843.

